# Treatment Strategies for Cutaneous and Oral Mucosal Side Effects of Oncological Treatment in Breast Cancer: A Comprehensive Review

**DOI:** 10.3390/biomedicines13081901

**Published:** 2025-08-04

**Authors:** Sanja Brnić, Bruno Špiljak, Lucija Zanze, Ema Barac, Robert Likić, Liborija Lugović-Mihić

**Affiliations:** 1 Department of Internal Medicine, Division of Oncology, Clinical Hospital Sveti Duh, 10000 Zagreb, Croatia; sanja.brnic@gmail.com; 2 Department of Oral Medicine, University of Zagreb School of Dental Medicine, 10000 Zagreb, Croatia; bspiljak@sfzg.unizg.hr; 3 Family Physician Office, 10000 Zagreb, Croatia; lucijazanze15@gmail.com (L.Z.); ema.barac@gmail.com (E.B.); 4 Department of Dermatovenereology, University Hospital Center Sestre Milosrdnice, 10000 Zagreb, Croatia; 5 Unit for Clinical Pharmacology, Department of Internal Medicine, Clinical Hospital Centre Zagreb, 10000 Zagreb, Croatia; robert.likic@mef.hr; 6 University of Zagreb School of Dental Medicine, 10000 Zagreb, Croatia

**Keywords:** breast cancer, cutaneous toxicity, oral mucosal toxicity, chemotherapy, targeted therapy, radiotherapy, immunotherapy, supportive care

## Abstract

Cutaneous and oral mucosal adverse events (AEs) are among the most common non-hematologic toxicities observed during breast cancer treatment. These complications arise across various therapeutic modalities including chemotherapy, targeted therapy, hormonal therapy, radiotherapy, and immunotherapy. Although often underrecognized compared with systemic side effects, dermatologic and mucosal toxicities can severely impact the patients’ quality of life, leading to psychosocial distress, pain, and reduced treatment adherence. In severe cases, these toxicities may necessitate dose reductions, treatment delays, or discontinuation, thereby compromising oncologic outcomes. The growing use of precision medicine and novel targeted agents has broadened the spectrum of AEs, with some therapies linked to distinct dermatologic syndromes and mucosal complications such as mucositis, xerostomia, and lichenoid reactions. Early detection, accurate classification, and timely multidisciplinary management are essential for mitigating these effects. This review provides a comprehensive synthesis of current knowledge on cutaneous and oral mucosal toxicities associated with modern breast cancer therapies. Particular attention is given to clinical presentation, underlying pathophysiology, incidence, and evidence-based prevention and management strategies. We also explore emerging approaches, including nanoparticle-based delivery systems and personalized interventions, which may reduce toxicity without compromising therapeutic efficacy. By emphasizing the integration of dermatologic and mucosal care, this review aims to support clinicians in preserving treatment adherence and enhancing the overall therapeutic experience in breast cancer patients. The novelty of this review lies in its dual focus on cutaneous and oral complications across all major therapeutic classes, including recent biologic and immunotherapeutic agents, and its emphasis on multidisciplinary, patient-centered strategies.

## 1. Introduction

Breast cancer remains the most commonly diagnosed malignancy among women worldwide and is one of the leading causes of cancer-related mortality, with over 2.3 million new cases reported annually. Despite advances in screening and early detection, the global incidence rates continue to rise [1,2,3]. In the past two decades, treatment strategies have significantly evolved to include cytotoxic chemotherapy, hormonal therapies, human epidermal growth factor receptor 2 (HER2)-targeted agents, cyclin-dependent kinase 4/6 (CDK4/6) inhibitors, immune checkpoint inhibitors (ICIs), and precision radiotherapy. These advancements have led to notable improvements in both progression-free and overall survival [4,5,6]. As survival rates improve, greater attention is being directed toward minimizing treatment-related toxicities and enhancing the patients’ quality of life throughout therapy and survivorship.

Among the most impactful side effects are mucocutaneous toxicities, which include a wide spectrum of dermatologic and oral mucosal complications. These toxicities are common across modern cancer therapies and encompass rashes, xerosis, pruritus, hand-foot syndrome, alopecia, mucositis, xerostomia, and pigmentary or nail changes [7,8]. Although often underrecognized compared with systemic complications, mucocutaneous toxicities are highly visible, potentially painful, and can significantly impair physical comfort, emotional well-being, social functioning, and adherence to treatment [9,10]. The likelihood, severity, and type of adverse events (AEs) frequently correspond to the mechanism of action of the therapeutic agent. For example, epidermal growth factor receptor (EGFR) and CDK4/6 inhibitors are linked to acneiform eruptions and alopecia; capecitabine is associated with hand-foot syndrome; and ICIs with immune-mediated mucocutaneous reactions. Oral toxicities, including mucositis, lichen planus-like lesions, dysgeusia, and opportunistic infections, are especially prevalent with targeted and immunotherapeutic agents but often remain underappreciated despite their clinical relevance [11,12].

The rise of precision oncology and biologically targeted therapies has further diversified the spectrum of mucocutaneous toxicities. These adverse effects are often dose-dependent, mechanism-specific, and in some cases predictable—allowing for risk stratification and anticipatory management. Recent studies have also highlighted the potential of nanoparticle-based delivery systems and biologic-modifying agents to reduce drug toxicity while maintaining therapeutic efficacy [13,14]. Optimal management requires early recognition, accurate classification, and multidisciplinary collaboration among oncologists, dermatologists, oral medicine specialists, nurses, and other healthcare professionals. This integrative approach can help prevent unnecessary dose modifications, alleviate psychological burden, and support treatment adherence—ultimately improving oncologic outcomes [15].

This narrative review provides a comprehensive synthesis of the current knowledge on cutaneous and oral mucosal toxicities associated with breast cancer therapies, with a focus on clinical presentation, underlying pathophysiology, incidence, and evidence-based management. Unlike prior reviews that examined these toxicities in isolation or across broader cancer populations, this review offers an integrated, breast cancer-specific perspective. It also explores emerging strategies—such as precision medicine, supportive care algorithms, and innovative drug delivery platforms—that reflect the future direction of oncology care. The novelty of this review lies in its dual focus on dermatologic and oral complications across all major therapeutic classes, and its emphasis on multidisciplinary, patient-centered approaches and novel management strategies.

## 2. Chemotherapy-Induced Cutaneous Toxicities

Chemotherapy remains a cornerstone of breast cancer treatment; however, it is commonly associated with a broad spectrum of cutaneous and mucosal toxicities that may significantly compromise the patients’ quality of life. These adverse effects are often dose-dependent, agent-specific, and temporally linked to treatment cycles, with severity ranging from mild discomfort to complications severe enough to warrant dose reduction or treatment interruption [16,17].

### 2.1. Alopecia

Chemotherapy-induced alopecia (CIA) is one of the most common and emotionally distressing side effects of cancer treatment, affecting approximately 65–100% of patients receiving anthracyclines or taxanes—the two most widely used and effective drug classes in breast cancer therapy for reducing recurrence and mortality [18,19,20,21,22]. These agents target rapidly proliferating cells, including hair matrix keratinocytes, resulting in diffuse, non-scarring anagen effluvium. Hair loss typically begins 7 to 14 days after treatment initiation and peaks around week 4 [23]. In many cases, it extends beyond the scalp, involving the eyebrows, eyelashes, and body hair, further intensifying its psychosocial impact [24,25]. While CIA is generally reversible, full regrowth may take 6 to 12 months, and permanent alopecia has been reported—especially following high-dose cyclophosphamide or busulfan treatment [22,26,27,28]. Telogen effluvium may also occur but is less frequent. Certain agents, such as EGFR inhibitors, may lead to hair fragility, slowed growth, or changes in texture rather than complete hair loss [8].

Scalp cooling systems (e.g., cold caps) remain the most effective preventive strategy. These devices induce localized vasoconstriction, thereby reducing the delivery of cytotoxic agents to hair follicles. Randomized controlled trials have shown success rates between 50% and 70%, particularly in taxane-only protocols [29,30,31,32]. Additionally, topical 5% minoxidil has proven effective in accelerating hair regrowth after chemotherapy; one trial demonstrated a reduction in regrowth time by approximately 1.5 months compared with the placebo [19,33]. Although minoxidil does not prevent initial hair loss, it provides benefits during the recovery phase. Other emerging strategies—such as cytokine modulation and the molecular targeting of follicular pathways—are currently being explored, though they remain experimental [19,34,35,36].

Patient education is crucial for preparing individuals for the possibility of hair loss and for supporting their emotional well-being. Counseling, the provision of wigs or head coverings, and reassurance about hair regrowth can greatly improve self-esteem and promote adherence to treatment.

### 2.2. Hand-Foot Syndrome

**Figure 1:**
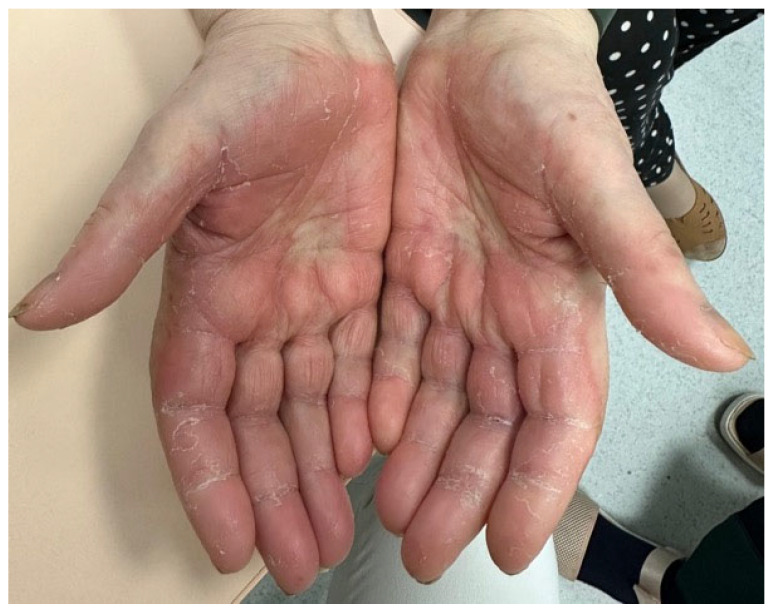
Palmar erythema with desquamation, consistent with hand-foot syndrome (palmoplantar erythrodysesthesia) in a patient undergoing capecitabine treatment (written informed consent was obtained from the patient for the publication of this clinical image).

Hand-foot syndrome (HFS), also known as palmar-plantar erythrodysesthesia, is a painful and potentially disabling cutaneous reaction associated with several chemotherapeutic agents including capecitabine, liposomal doxorubicin, and 5-fluorouracil (Figure 1). Clinically, it is characterized by well-demarcated erythema, edema, dysesthesia, and desquamation of the palms and soles. These may be accompanied by tingling, swelling, hyperkeratosis, blistering, or ulceration, all of which can significantly impair daily activities such as walking, grasping objects, or typing [37,38,39].

The underlying pathogenesis involves direct cytotoxicity to rapidly proliferating epidermal keratinocytes, particularly in pressure- and friction-exposed acral areas. Localized drug accumulation—attributed to vascular distribution and high eccrine gland density—likely contributes to the development of HFS. In some cases, related reactions (collectively termed toxic erythema of chemotherapy) may extend to intertriginous areas such as the axillae, groin, and popliteal fossae [38,40].

Severity grading based on the Common Terminology Criteria for Adverse Events (CTCAE v5.0) provides a standardized framework for classification and management [41]:

- Grade 1 (mild): Minimal skin changes (e.g., erythema, tingling) without pain or functional impairment;
- Grade 2 (moderate): Painful erythema and desquamation interfering with daily activities; may require temporary dose adjustment;
- Grade 3 (severe): Blistering, ulceration, or disabling pain necessitating dose reduction or treatment discontinuation.

In clinical studies, grade ≥2 HFS has been reported in up to 60% of patients treated with capecitabine, especially at cumulative doses exceeding 1250 mg/m^2^ twice daily for more than 14 days [42,43]. Symptoms typically arise after the first or second treatment cycle and often worsen cumulatively. A wide range of agents have been associated with HFS including cyclophosphamide, cytarabine, daunorubicin, docetaxel, etoposide, fluorouracil, hydroxyurea, idarubicin, methotrexate, paclitaxel, sorafenib, sunitinib, and vinblastine, among others [44].

Management involves both preventive strategies and symptomatic control. Patients should be advised to avoid heat, friction, and pressure and to apply urea-based emollients regularly to maintain skin hydration and reduce keratinocyte stress. In moderate to severe cases, topical corticosteroids can reduce inflammation, while systemic analgesics may help alleviate pain [38,44]. Cold compresses, non-adherent dressings, and wound care can provide additional relief. In refractory or grade 3 cases, chemotherapy dose reduction, treatment delay, or discontinuation may be necessary. Clinical resolution generally occurs within 14 to 28 days [38,44,45].

A proactive, preventive approach is crucial—particularly in patients receiving capecitabine or liposomal anthracyclines—where early intervention may preserve treatment continuity and improve quality of life.

### 2.3. Erythema Exudativum Multiforme (EEM)

**Figure 2:**
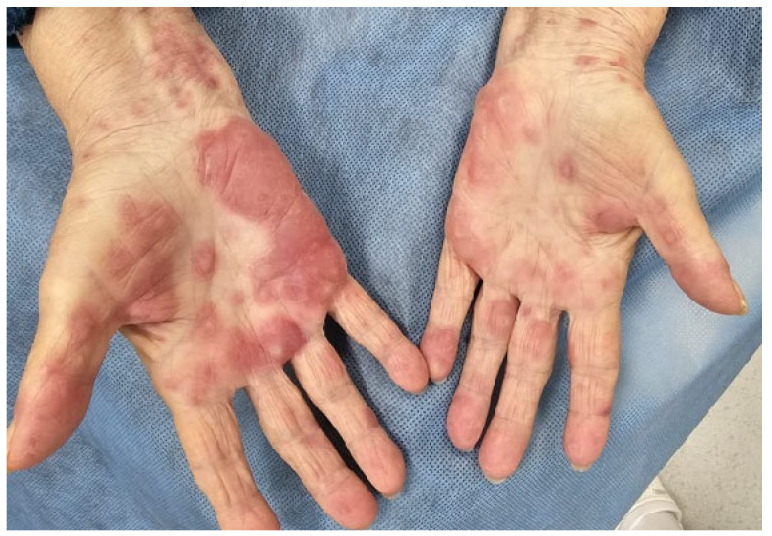
Erythema exudativum multiforme involving the palms, characterized by targetoid and dusky red lesions, observed following adjuvant chemotherapy (written informed consent was obtained from the patient for the publication of this clinical image).

Erythema exudativum multiforme (EEM) is a rare but clinically significant inflammatory dermatosis that may be triggered by certain chemotherapeutic agents, particularly platinum-based compounds and antimetabolites. Clinically, EEM is characterized by classic targetoid lesions—symmetrical, round, erythematous macules or papules with a central dusky zone and a surrounding pale halo—most commonly affecting the extensor surfaces, palms, and soles. In patients with breast cancer, EEM has been reported as a cutaneous adverse reaction following adjuvant chemotherapy [46,47] (Figure 2). Although generally self-limited, the condition can cause considerable discomfort and cosmetic concern.

The diagnosis is primarily clinical but may be supported by histopathological findings on skin biopsy, typically revealing interface dermatitis with necrotic keratinocytes and a perivascular lymphocytic infiltrate [46,47]. Management involves prompt discontinuation of the suspected causative agent and supportive care. Depending on the severity, patients may benefit from topical corticosteroids in mild cases or systemic corticosteroids in more extensive disease. Early recognition is crucial to prevent progression to more severe mucocutaneous reactions such as Stevens–Johnson syndrome (SJS) [48,49,50,51].

### 2.4. Nail and Skin Changes

Nail and skin toxicities are common, visible, and often distressing adverse effects of chemotherapy in breast cancer patients, ranking third in prevalence after hair and skin reactions [16]. These complications result from the cytotoxic effects of antineoplastic agents on rapidly proliferating epithelial cells of the nail matrix and epidermis. The incidence and severity vary depending on the chemotherapeutic regimen, with taxanes (e.g., docetaxel, paclitaxel) and anthracyclines (e.g., doxorubicin, epirubicin) being the most frequently implicated agents [52,53,54]. Other contributors include capecitabine, hydroxyurea, and EGFR inhibitors [55,56,57].

**Figure 3:**
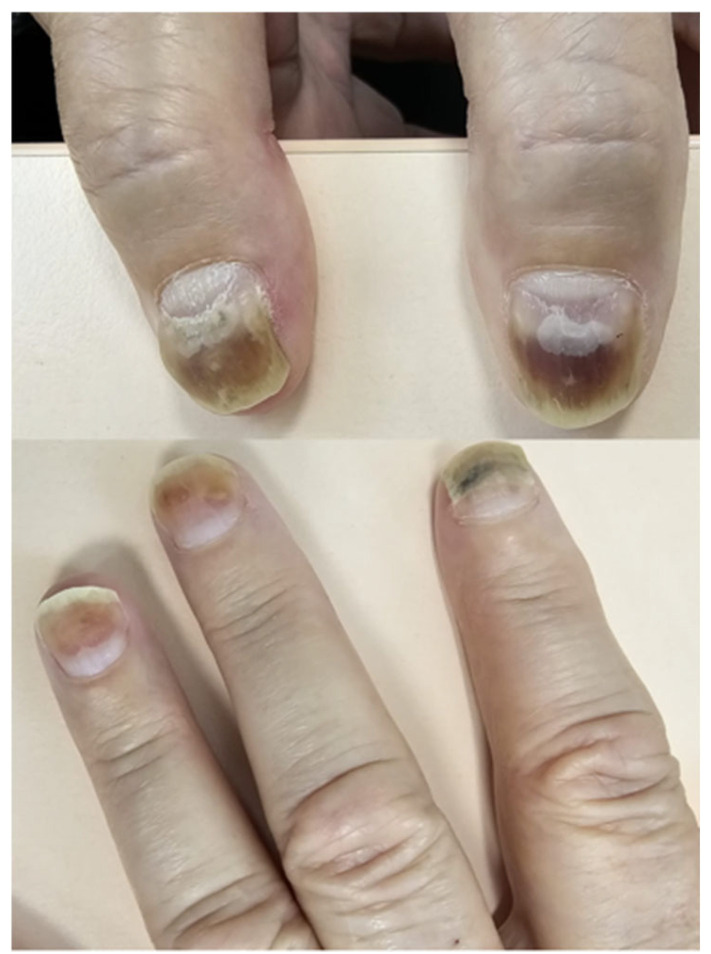
Onychomadesis with subungual hemorrhages, manifesting as nail plate separation and discoloration in a patient treated with a combination of anthracyclines and taxanes (written informed consent was obtained from the patient for the publication of this clinical image).

**Figure 4:**
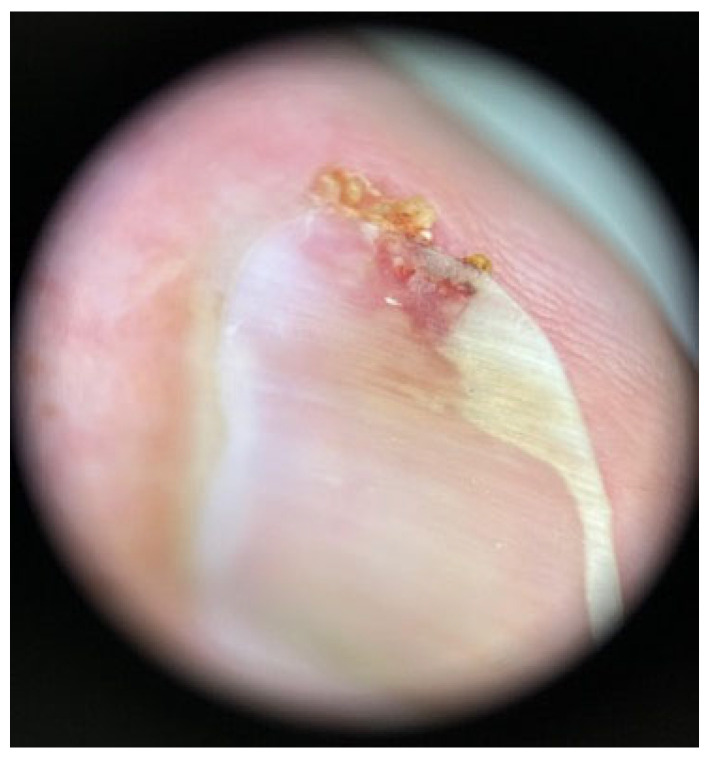
Periungual pyogenic granuloma localized at the proximal nail fold, presenting with friable vascular tissue, induced by taxane-based chemotherapy (written informed consent was obtained from the patient for the publication of this clinical image).

Nail changes encompass a broad spectrum of abnormalities [58] (Figure 3 and Figure 4). Common manifestations include onycholysis (separation of the nail plate from the nail bed), onychomadesis (complete nail shedding), Beau’s lines (transverse grooves), leukonychia (white discoloration), melanonychia (nail pigmentation), and paronychia (inflammation of the nail folds) [55,57]. These alterations may cause discomfort, pain, or functional impairment, especially when multiple digits are affected. EGFR inhibitors are particularly associated with paronychia, which occurs in up to 10–15% of patients, while the incidence is lower (<1%) with capecitabine [55,59,60]. Although rarely dangerous, the cosmetic and functional consequences can be significant. Most nail abnormalities are self-limiting and gradually resolve as the nail grows out after treatment cessation [52,55].

Skin toxicities, especially xerosis (dry skin), are also frequently observed. Prevalence varies by treatment type and individual patient factors, ranging from 4.4% to over 90% in reported studies [16,61,62]. Xerosis may lead to pruritus, scaling, fissuring, and an increased risk of secondary infections [63,64]. It commonly affects the hands and feet but can also involve other body areas. The pathophysiology includes direct epithelial damage, impaired skin barrier function, and reduced sebaceous gland activity caused by cytotoxic therapies.

Management of nail and skin toxicities is primarily supportive, aiming to prevent complications and relieve symptoms. Preventive strategies for nail damage include the use of cryotherapy gloves during taxane infusion to reduce local drug exposure to the nail matrix [65,66]. Patients should be advised on gentle nail care practices—such as keeping nails short, avoiding trauma, and refraining from nail polish or artificial nails. Paronychia may be treated with topical or systemic antibiotics (e.g., doxycycline) and analgesics; more severe cases may require antiseptic soaks or surgical drainage [63,67]. For painful nail bed exposure, non-adherent dressings can offer protection.

Xerosis should be managed with the daily application of emollients—particularly those containing urea or ceramides—and the avoidance of irritants such as harsh soaps and prolonged water exposure. Barrier creams and cotton gloves, especially when worn overnight, can help restore hydration and support skin healing [63]. Though simple, these interventions are essential for maintaining patient comfort, reducing secondary complications, and promoting adherence to cancer therapy.

### 2.5. Photosensitivity

**Figure 5:**
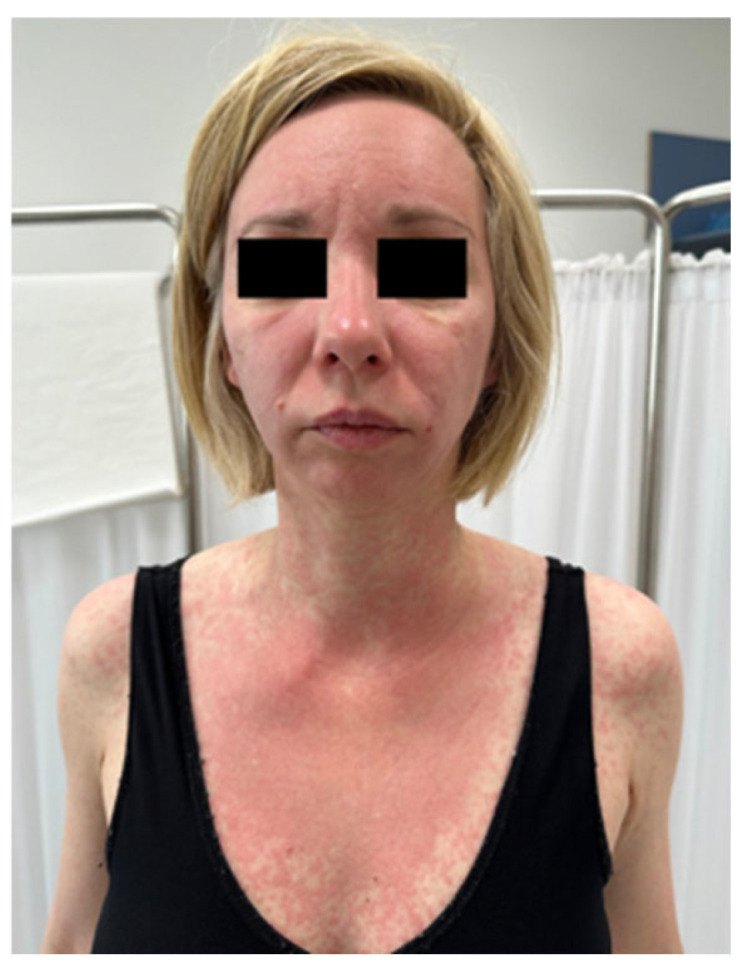
Phototoxic reaction presenting as sharply demarcated erythema and maculopapular rash in sun-exposed areas following fluorouracil-based chemotherapy (written informed consent was obtained from the patient for the publication of this clinical image).

Photosensitivity is a moderately common adverse reaction to certain chemotherapeutic agents, characterized by an increased sensitivity to ultraviolet (UV) radiation. Clinically, it presents as exaggerated sunburn or erythematous skin reactions following minimal sun exposure. The agents most frequently associated with photosensitivity include methotrexate, fluorouracil, and dacarbazine [68,69] (Figure 5).

Prevention is essential, and patients should be routinely counseled on photoprotection strategies. These include the daily use of broad-spectrum sunscreens with high sun protection factor (SPF), wearing UV-protective clothing and wide-brimmed hats, and avoiding direct sunlight—particularly during peak UV radiation hours. Proactive measures can substantially reduce the risk of severe photosensitive reactions and help maintain patient comfort during chemotherapy [68,69].

### 2.6. Hyperpigmentation

Hyperpigmentation is a well-recognized cutaneous manifestation associated with certain chemotherapeutic agents. One of the most distinctive patterns is flagellate hyperpigmentation induced by bleomycin [8,70,71]. It appears as dark brown linear streaks, typically around 10 cm in length, arranged in a crisscross or whip-like pattern resembling a flagellum. Although the exact pathogenesis remains unclear, it is hypothesized that bleomycin induces pruritus, particularly on the trunk, prompting scratching behavior. This mechanical irritation may lead to local drug accumulation and subsequent pigmentary changes in the affected areas [8,70,71].

In addition to bleomycin, several other chemotherapeutic agents—including fluorouracil, vinorelbine, and daunorubicin—can cause diffuse or localized hyperpigmentation of the skin, nails, or oral mucosa [11,72]. These changes are typically non-flagellate and may present as patchy macules or follow venous distribution patterns, depending on drug metabolism and route of administration [73].

Management of chemotherapy-induced hyperpigmentation is primarily supportive. Topical depigmenting agents, such as hydroquinone, may help lighten affected areas by reducing melanin synthesis. In cases accompanied by pruritus, oral antihistamines can provide symptomatic relief. Most pigmentary changes gradually resolve after discontinuation of the causative agent, although complete resolution may take several weeks to months [8,70,71,72,73].

### 2.7. Radiation-Recall Dermatitis

Radiation-recall dermatitis is an acute inflammatory skin reaction that occurs in areas previously exposed to radiation therapy or severe sunburn, typically triggered by the administration of certain chemotherapeutic agents. This phenomenon may arise weeks to months after the initial radiation exposure. Clinically, it presents as erythema, edema, or desquamation localized to previously irradiated skin, despite an intervening asymptomatic period. Although the exact mechanism is not fully understood, it is hypothesized to involve the reactivation of subclinically damaged keratinocytes, which are particularly vulnerable due to their high proliferative capacity [74].

Chemotherapeutic agents most frequently associated with recall reactions include gemcitabine, methotrexate, docetaxel, etoposide, and doxorubicin [52,54,74]. Management focuses on minimizing further UV exposure, initiating appropriate wound care, and applying topical corticosteroids to reduce inflammation. Patient counseling is essential; individuals should be advised to allow for complete recovery of the irradiated skin before starting chemotherapy in order to reduce the risk of this adverse reaction.

### 2.8. Skin Necrosis

Skin necrosis refers to the localized death of skin tissue, clinically presenting as blackened, devitalized areas that may subsequently slough off. This condition most often results from direct toxic injury, typically following extravasation—the accidental leakage of intravenously administered chemotherapeutic agents into the surrounding subcutaneous tissue.

Chemotherapeutic agents are broadly classified based on their potential to cause tissue injury into irritants and vesicants. Irritants (e.g., certain taxanes and platinum compounds) may induce phlebitis, pain, and chemical cellulitis. Vesicants, such as doxorubicin, pose a greater risk and can cause extensive necrosis, ulceration, and even thrombosis of local vasculature [61,75,76]. Doxorubicin is a well-known prototype of vesicant agents capable of causing significant tissue destruction upon extravasation.

Management involves immediate and thorough local wound care including the use of cold or warm compresses depending on the agent involved [75,76,77]. Early consultation with a plastic surgeon is recommended in cases of vesicant extravasation—particularly involving anthracyclines—due to the risk of progressive necrosis, which may require surgical debridement or reconstructive procedures.

### 2.9. Neutrophilic Eccrine Hidradenitis

Neutrophilic eccrine hidradenitis (NEH) is a rare cutaneous reaction that typically presents as tender, erythematous papules, plaques, or nodules localized to the trunk, face, and ears. The underlying pathogenesis is believed to involve the accumulation of high concentrations of chemotherapeutic agents—most notably cytarabine and bleomycin—within eccrine sweat glands, leading to localized neutrophilic inflammation [78,79]. Diagnosis is confirmed via skin biopsy, which reveals a characteristic neutrophilic infiltrate surrounding the eccrine coils, sometimes accompanied by necrosis of the eccrine epithelium.

Although NEH is usually self-limiting and resolves spontaneously within several days to weeks, symptomatic treatment may be beneficial. Systemic corticosteroids, nonsteroidal anti-inflammatory drugs (NSAIDs), and dapsone have been used to shorten the disease duration and relieve discomfort in more symptomatic cases [78,79]. Prompt recognition is important to avoid the unnecessary discontinuation of chemotherapy and to ensure appropriate supportive management.

### 2.10. Eccrine Squamous Metaplasia (Syringometaplasia)

Eccrine squamous metaplasia, also known as syringometaplasia, is a rare cutaneous adverse reaction involving the upper portion of eccrine sweat ducts. Clinically, it presents as nonspecific erythematous plaques or crusted papular eruptions, sometimes mimicking infectious or inflammatory dermatoses. A distinct variant affects intertriginous regions, such as the axillae, groin, and lateral neck, areas commonly exposed to friction and moisture.

This condition may be triggered by three major classes of chemotherapeutic agents: (1) nitrogen mustards including cyclophosphamide, chlorambucil, and melphalan; (2) anthracyclines such as doxorubicin, idarubicin, and epirubicin; and (3) antimetabolites including azathioprine, methotrexate, fluorouracil, and capecitabine [61,80,81,82,83].

Eccrine squamous metaplasia typically resolves spontaneously without long-term sequelae, and treatment is generally supportive, similar to that for neutrophilic eccrine hidradenitis. However, recurrence occurs in approximately 50% of patients upon re-exposure to the same chemotherapeutic agent, emphasizing the importance of careful monitoring and the documentation of prior reactions [80,81,82,83].

### 2.11. Sclerotic Dermal Reactions

Sclerotic dermal reactions are rare but clinically significant cutaneous complications that may arise during chemotherapy with agents such as bleomycin and docetaxel. These reactions are characterized by scar-like skin induration, often mimicking localized scleroderma (morphea) or, in more extensive cases, resembling features of systemic sclerosis [84,85,86].

Although the exact pathogenesis is not fully understood, it is hypothesized that these agents promote fibroblast activation and collagen overproduction, resulting in dermal thickening and sclerosis [84,85,86,87,88]. Affected skin areas may appear firm, hypopigmented, and shiny, with reduced elasticity that can impair mobility depending on the site of involvement.

While most cases are self-limiting, resolution typically occurs only after discontinuation of the causative agent. Early recognition is essential to prevent progression and to differentiate these reactions from other fibrosing disorders. In persistent or symptomatic cases, dermatologic consultation may be warranted, and treatment may include topical corticosteroids or physical therapy to alleviate discomfort and preserve skin flexibility [84,85,86,87,88].

### 2.12. Raynaud’s Phenomenon

Raynaud’s phenomenon is a vasospastic disorder characterized by transient, sharply demarcated discoloration of the digits—typically progressing from white (ischemia), to blue (deoxygenation), and finally to red (reperfusion)—in response to cold exposure or emotional stress. This condition may be induced or exacerbated by several chemotherapeutic agents including bleomycin, cisplatin, gemcitabine, and rituximab [89,90].

In contrast, cutaneous vasculitis involves the immune-mediated inflammation of blood vessel walls, leading to vascular occlusion, tissue ischemia, and in severe cases, necrosis. Clinically, vasculitis may present with livedo reticularis, ulcerations, palpable purpura, or digital infarcts. Although Raynaud’s phenomenon and vasculitis may share overlapping features, they differ significantly in pathophysiology and require distinct diagnostic approaches.

In suspected cases of vasculitis, skin biopsy is essential for confirming the diagnosis and excluding other etiologies. Management involves discontinuation of the offending agent and the initiation of systemic corticosteroids at immunosuppressive doses. Supportive care for Raynaud’s phenomenon includes thermal protection (e.g., hand warmers, gloves) and lifestyle modifications to avoid cold exposure. In moderate to severe cases, vasodilatory pharmacotherapy, such as calcium channel blockers or angiotensin-converting enzyme (ACE) inhibitors, may help improve peripheral perfusion and relieve symptoms [89,90,91].

Both conditions typically improve after cessation of the causative chemotherapeutic agent; however, persistent or recurrent cases may require long-term vascular or immunosuppressive management.

In summary, early recognition and accurate grading of chemotherapy-induced dermatologic toxicities are essential for guiding dose modifications, initiating timely supportive care, and ensuring treatment adherence. Failure to proactively manage these adverse events may compromise not only patient comfort, but also the overall oncologic outcomes.

## 3. Targeted Therapy-Induced Dermatologic Effects

Targeted therapies have transformed breast cancer management by selectively inhibiting molecular pathways essential for tumor growth and survival. Although generally better tolerated than conventional chemotherapy, these agents are frequently associated with distinctive dermatologic toxicities. The nature and severity of cutaneous side effects vary depending on the specific molecular target and mechanism of action, with the EGFR, HER2, and phosphoinositide 3-kinase–AKT–mechanistic target of rapamycin (PI3K–AKT–mTOR) signaling pathways being particularly implicated [92,93,94,95,96].

### 3.1. EGFR and HER2 Inhibitors

Lapatinib, a dual tyrosine kinase inhibitor targeting both the EGFR and HER2, is commonly used in the treatment of HER2-positive breast cancer. Inhibition of EGFR signaling, which plays a critical role in epidermal homeostasis, results in various mucocutaneous toxicities, most notably xerosis, pruritus, and acneiform eruptions [63,97,98].

#### 3.1.1. Xerosis and Mucosal Involvement

Xerosis (dry skin) is among the most frequent dermatologic adverse effects of EGFR inhibitors and often presents with greasy scaling resembling seborrheic dermatitis. The underlying pathogenesis involves the EGFR blockade-induced disruption of keratinocyte proliferation and premature terminal differentiation, resulting in compromised stratum corneum integrity. This barrier dysfunction may also predispose patients to mucosal involvement, potentially affecting the oral, ocular, and vaginal mucosae [97,98].

#### 3.1.2. Pruritus

Pruritus is frequently underreported but may occur independently or in association with xerosis or acneiform eruptions. The mechanism is believed to involve barrier disruption and increased transepidermal water loss, leading to sensitization of the cutaneous sensory nerve endings [97,98,99]. Severity varies, but chronic pruritus can significantly impair the quality of life and sleep. Management includes the regular use of emollients, topical corticosteroids, or calcineurin inhibitors, with systemic antihistamines or gabapentinoids considered in more severe or persistent cases [97,98].

#### 3.1.3. Acneiform Eruptions

Acneiform rash, also referred to as folliculitis, typically presents as sterile papules and pustules on the face, upper chest, and back. Unlike acne vulgaris, comedones are absent. This reaction results from inflammation of the follicular epithelium following EGFR inhibition. Implicated agents include actinomycin D, gefitinib, cetuximab, and lapatinib [98,100,101,102].

Prophylactic oral tetracyclines, such as doxycycline (100 mg twice daily), have been shown to reduce the severity of the rash. Adjunctive measures include topical corticosteroids to manage inflammation, emollients to support barrier repair, and broad-spectrum sunscreens to provide photoprotection. In severe or refractory cases, temporary dose modification or treatment interruption may be warranted [63,97,98,99,100,101,102].

Early recognition and prompt management are essential to prevent dose-limiting toxicity and maintain treatment adherence.

### 3.2. PI3K Inhibitors

Alpelisib, a selective phosphoinositide 3-kinase alpha (*PI3Kα*) inhibitor approved for hormone receptor-positive, HER2-negative, phosphatidylinositol-4,5-bisphosphate 3-kinase catalytic subunit alpha (*PIK3Cα*)-mutated breast cancer, is frequently associated with dermatologic adverse effects. The most common cutaneous toxicity is a maculopapular rash, typically appearing within 10 days of treatment initiation [103,104,105,106]. The rash commonly affects the trunk and extremities and may be accompanied by pruritus.

Mild rashes are generally managed with oral antihistamines and low-potency topical corticosteroids. In more severe cases (grade ≥2), particularly those accompanied by systemic symptoms such as fever, treatment with systemic corticosteroids (e.g., prednisone 0.5–1 mg/kg) is recommended, alongside temporary interruption of alpelisib therapy [103,104,105,106,107,108]. Regular dermatologic monitoring, especially during the first month of treatment, is advised to ensure early detection and the appropriate management of skin reactions.

### 3.3. Precision Oncology and Predictive Markers

The integration of genomic profiling has enabled the development of precision therapies targeting HER2, PI3K, CDK4/6, and immune checkpoints in breast cancer. However, these innovations are also associated with a range of emerging mucocutaneous toxicities—some of which may be underrecognized, idiosyncratic, or delayed in onset [109,110,111]. As previously noted, certain genotypes, such as *PIK3Cα* mutations in patients receiving alpelisib, have been linked to an increased risk of dermatologic adverse events [103,104,105,106,107,108].

**Figure 6:**
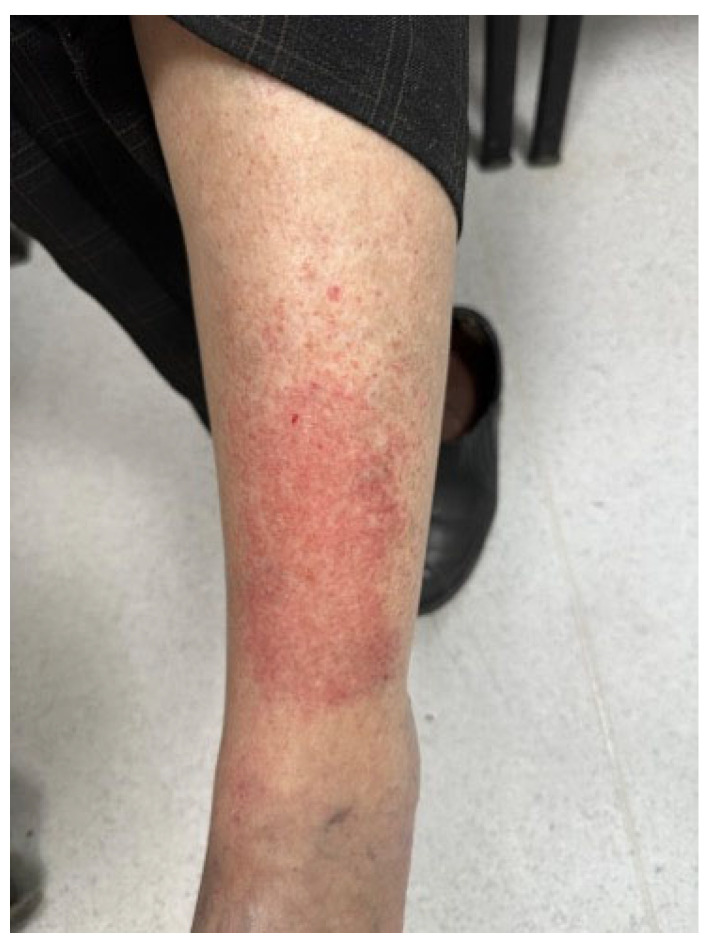
Erythematous and scaly skin lesions of the lower leg observed in a breast cancer patient receiving combination therapy with ribociclib (CDK4/6 inhibitor) and letrozole (aromatase inhibitor). The reaction was consistent with a drug-induced dermatitis (written informed consent was obtained from the patient for the publication of this clinical image).

**Figure 7:**
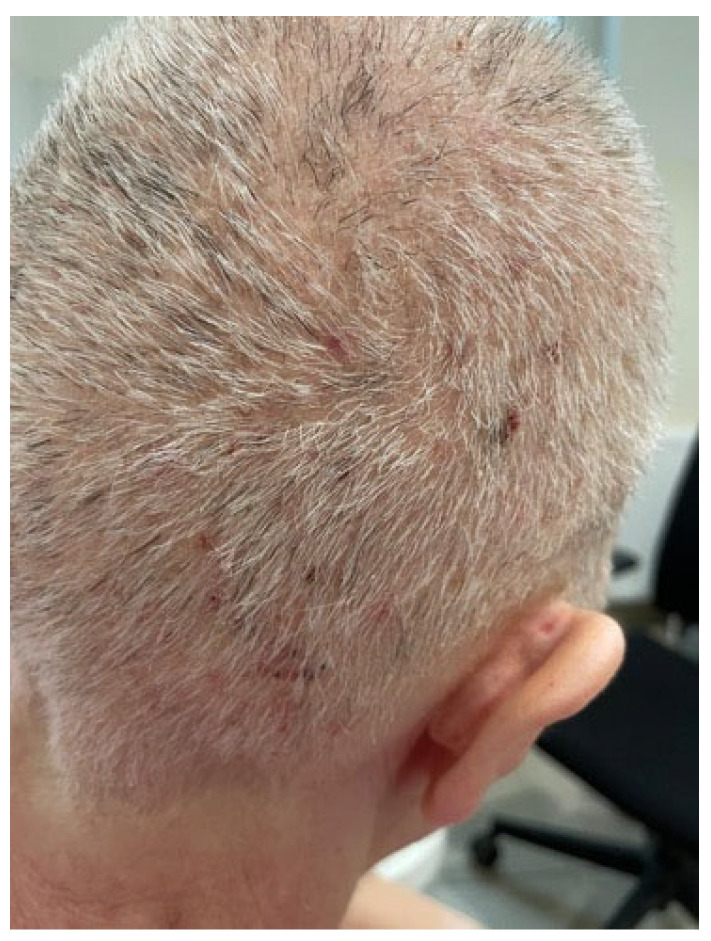
Folliculitis-like papular eruption on the scalp, composed of erythematous papules and pustules, in a patient receiving ribociclib (CDK4/6 inhibitor) and letrozole (aromatase inhibitor) (written informed consent was obtained from the patient for the publication of this clinical image).

Additionally, CDK4/6 inhibitors (e.g., palbociclib, ribociclib) have been associated with psoriasiform, papular, and eczematous eruptions, particularly in individuals with pre-existing atopy or psoriasis [112,113,114] (Figure 6 and Figure 7).

The emerging field of pharmacogenomics has provided deeper insights into individual susceptibility to mucocutaneous toxicity. For instance, polymorphisms in CYP2D6, a key enzyme in tamoxifen metabolism, can influence both the efficacy and toxicity, potentially affecting cutaneous and mucosal tolerance. Similarly, UGT1A1*28 variants, which impair glucuronidation of irinotecan metabolites, may lead to increased systemic exposure and heighten the risk of toxic erythema or oral mucositis.

Other genetic variants involved in immune regulation (e.g., specific HLA alleles) or epithelial integrity may predispose patients to autoimmune-like skin eruptions or lichenoid mucositis during immunotherapy or targeted therapies [115,116].

Although pharmacogenomic testing is not yet standard practice for predicting dermatologic toxicity, it holds promise for molecular risk stratification. This could support personalized, preemptive interventions such as enhanced clinical surveillance or prophylactic skin care regimens in high-risk individuals [116]. As precision oncology continues to advance, close collaboration with dermatologists and oral medicine specialists within multidisciplinary care teams is crucial for optimizing patient outcomes. Clinicians should remain vigilant for atypical or delayed mucocutaneous reactions and should proactively educate patients about early warning signs warranting timely dermatologic or oral referral.

## 4. Hormonal Therapy-Associated Skin Effects

Hormonal therapies, including aromatase inhibitors (AIs; e.g., letrozole, anastrozole) and selective estrogen receptor modulators (SERMs; e.g., tamoxifen), are fundamental in the treatment of hormone receptor-positive breast cancer. By blocking or modulating estrogen signaling, these agents substantially reduce recurrence risk and improve survival outcomes [117,118,119]. However, estrogen deprivation may give rise to a range of mucocutaneous adverse effects including alopecia, xerosis, vulvovaginal atrophy, and more rarely, autoimmune-mediated dermatoses [63,117,118,119].

### 4.1. Alopecia and Xerosis

Estrogen plays a key role in maintaining hair follicle integrity and skin hydration. Both AIs and SERMs disrupt this hormonal balance, leading to epidermal thinning, reduced sebaceous gland activity, and hair loss resembling an androgenic pattern. Unlike the abrupt hair loss associated with chemotherapy, hormone therapy-related alopecia typically presents as diffuse thinning—particularly over the midline and crown—resembling female-pattern hair loss [120,121].

Management strategies include topical 5% minoxidil, which has demonstrated efficacy in stimulating hair regrowth. In selected patients, low-level laser therapy may also be considered, although supporting evidence remains limited.

Xerosis is another common cutaneous side effect of hormonal therapy, presenting as generalized dryness, flaking, and pruritus—especially over the extremities. Recommended management includes the twice-daily application of ceramide-based or urea-containing emollients, particularly after bathing. Patients should be advised to avoid prolonged exposure to hot water, harsh soaps, and sun, all of which can exacerbate epidermal barrier dysfunction [63,122].

### 4.2. Vulvovaginal Atrophy and Genitourinary Syndrome

Genitourinary syndrome of menopause (GSM)—encompassing vulvovaginal atrophy, dryness, dyspareunia, and urinary symptoms—is a prevalent but often underreported complication in patients receiving AIs or SERMs. These symptoms can severely affect sexual function, emotional well-being, and treatment adherence [123,124,125].

First-line management includes non-hormonal interventions such as vaginal moisturizers, lubricants, and hyaluronic acid-based products, which typically offer substantial symptomatic relief. In more severe or refractory cases, topical lidocaine may alleviate burning sensations during intercourse or routine activities.

For patients with persistent symptoms, and following careful oncologic consultation, the judicious use of ultra-low-dose vaginal estrogen or dehydroepiandrosterone (DHEA) may be considered. Fractional CO_2_ laser therapy is also emerging as a promising non-hormonal treatment modality, with growing evidence supporting its safety and efficacy without systemic estrogen exposure [123,124,125,126,127].

### 4.3. Immune-Mediated and Rare Cutaneous Reactions

Although uncommon, SERMs and AIs have been implicated in immune-mediated skin reactions such as urticarial vasculitis, subacute cutaneous lupus erythematosus (SCLE), and lichen planus-like eruptions [128,129,130,131]. These conditions often present as pruritic, erythematous plaques over sun-exposed or intertriginous areas and may be accompanied by systemic symptoms such as arthralgia or low-grade fever.

Prompt dermatologic assessment is essential to differentiate these reactions from paraneoplastic dermatoses or infections. Treatment typically involves discontinuation of the offending agent and the initiation of systemic corticosteroids, antimalarials (e.g., hydroxychloroquine), or immunosuppressive therapy depending on the clinical severity [128,129,130,131].

### 4.4. Clinical Considerations

While most mucocutaneous adverse effects of hormonal therapy are manageable, they can negatively impact long-term treatment adherence. Clinicians should proactively screen for symptoms and implement early interventions to minimize discomfort and avoid premature treatment discontinuation. A multidisciplinary approach, including dermatology, gynecology, and oncology, is vital to balance therapeutic efficacy with quality-of-life preservation [118,132].

## 5. Radiotherapy-Induced Cutaneous Reactions

Radiotherapy is a cornerstone in the management of breast cancer, particularly in early-stage and locoregionally advanced disease. Despite its therapeutic benefits, radiotherapy is almost invariably associated with cutaneous adverse effects, collectively referred to as radiodermatitis. These reactions are typically classified as either acute or chronic, based on their time of onset and underlying pathophysiological mechanisms [133,134].

Acute radiodermatitis generally develops within the first one to four weeks of treatment and results from radiation-induced damage to rapidly proliferating basal keratinocytes. Clinically, it presents as erythema, xerosis (dryness), pruritus, and desquamation, which may be dry or moist depending on the extent of epidermal injury. In more severe cases, patients may experience edema, discomfort, bullae formation, or ulceration, potentially necessitating treatment interruptions and adversely affecting quality of life [133,134,135,136]. The severity and trajectory of acute skin reactions are influenced by several factors including the total radiation dose, fractionation schedule, concurrent chemotherapy (particularly taxanes), skin phototype, and comorbidities such as diabetes, obesity, or smoking [133,134,135,136,137,138,139].

Chronic radiodermatitis may manifest months to years after the completion of therapy and is characterized by cumulative damage to the dermal vasculature, fibroblasts, and adnexal structures. Clinical features include persistent skin atrophy, telangiectasia, fibrosis, and pigmentary changes such as hyperpigmentation, hypopigmentation, or mottled discoloration [133,140,141]. In rare instances, chronic complications may involve non-healing ulcers, cutaneous necrosis, or secondary malignancies such as radiation-induced angiosarcoma [140,141,142,143].

Standardized grading of radiotherapy-induced skin toxicity is essential for clinical assessment and therapeutic decision-making. The most widely used classification systems include:

- CTCAE v5.0:
  ○ Grade 1: Faint erythema or dry desquamation;
  ○ Grade 2: Moderate to brisk erythema; patchy moist desquamation, mainly in skin folds;
  ○ Grade 3: Moist desquamation outside of skin folds; bleeding induced by minor trauma;
  ○ Grade 4: Full-thickness dermal ulceration or necrosis; spontaneous bleeding;
  ○ Grade 5: Death (extremely rare) [41].
- Radiation Therapy Oncology Group (RTOG) Acute Radiation Morbidity Scoring Criteria:
  ○ Grade 0: No visible change;
  ○ Grade 1: Follicular erythema, epilation, dry desquamation;
  ○ Grade 2: Patchy moist desquamation, moderate edema;
  ○ Grade 3: Confluent moist desquamation, pitting edema;
  ○ Grade 4: Ulceration, hemorrhage, necrosis [144].

These frameworks facilitate consistent documentation, inform supportive care protocols, and guide decisions regarding dose adjustments or treatment breaks.

The cornerstone of management is preventive care, initiated prior to radiotherapy and maintained throughout its course. Patients should be counseled on gentle skin care practices including the use of mild, pH-neutral cleansers, avoidance of abrasive clothing or adhesives over the treated area, and consistent sun protection. Non-irritating moisturizers—particularly those containing hyaluronic acid or calendula extract—have demonstrated efficacy in maintaining skin hydration and mitigating acute symptoms [135,145,146].

For mild to moderate reactions, mid-potency topical corticosteroids, such as mometasone furoate or betamethasone valerate, may reduce erythema, pruritus, and discomfort, and may delay progression to moist desquamation [133,147,148]. Moist desquamation requires meticulous wound care including the application of non-adherent dressings, and when necessary, topical antimicrobials such as silver sulfadiazine to prevent secondary infection.

Emerging therapies, including barrier-forming films, topical sucralfate, vitamin D analogs, and low-level laser therapy, are currently under investigation and may offer additional benefit in selected cases [149,150].

In chronic radiodermatitis, management remains largely supportive. Fibrosis may respond to physical therapy, massage, or topical agents such as pentoxifylline and tocopherol, although evidence is limited. Telangiectasia and pigmentary alterations are usually permanent but may be treated cosmetically using vascular laser technologies [136,137,140,141,146].

Optimal care requires a multidisciplinary approach, involving oncologists, dermatologists, wound care specialists, and radiation therapists. Patient education, early recognition of evolving skin changes, and timely interventions are essential to preserve treatment adherence and minimize long-term sequelae.

## 6. Immunotherapy-Related Dermatologic Effects

The advent of ICIs, such as atezolizumab (anti-PD-L1), has significantly improved the outcomes in select breast cancer subtypes, particularly triple-negative breast cancer (TNBC). These agents function by inhibiting immune checkpoints, including PD-1/PD-L1 and CTLA-4 pathways, thereby enhancing T-cell-mediated antitumor responses.

However, the reactivation of immune surveillance is nonspecific and may precipitate immune-related adverse events (irAEs), with the skin and mucous membranes being among the most frequently involved sites [151,152,153,154,155,156,157].

Cutaneous irAEs typically emerge within the first few weeks to months of therapy and often represent the earliest and most visible manifestations of immune dysregulation. Clinical presentations span a broad spectrum—from mild, self-limiting rashes to severe, potentially life-threatening reactions. Common manifestations include maculopapular eruptions, lichenoid dermatitis, pruritus, and psoriasiform eruptions [158,159,160,161,162,163]. Less frequently, patients may develop vitiligo-like depigmentation (most commonly in melanoma, though also reported in breast cancer) or autoimmune blistering disorders such as bullous pemphigoid [164,165,166,167,168,169].

The underlying mechanism involves the aberrant activation of T cells with cross-reactivity against skin-associated self-antigens. The histopathologic analysis of skin biopsies often demonstrates interface dermatitis or perivascular lymphocytic infiltrates, consistent with autoimmune-mediated damage [157,161,170,171,172,173].

Management of cutaneous irAEs is stratified according to severity, using the CTCAE v5.0:

- Grade 1 (Mild): Limited skin involvement without significant symptoms.
  ○ Management: Topical corticosteroids (e.g., hydrocortisone, mometasone) and oral antihistamines for pruritus.
- Grade 2 (Moderate): Widespread involvement or bothersome pruritus.
  ○ Management: Temporary suspension of immunotherapy, initiation of medium- to high-potency topical corticosteroids.
- Grade 3–4 (Severe): Extensive involvement, ulceration, or bullous lesions (e.g., Stevens-Johnson syndrome, toxic epidermal necrolysis).
  ○ Management: Immediate discontinuation of ICI therapy, systemic corticosteroids (e.g., prednisone 1–2 mg/kg/day), and often inpatient dermatologic care. In refractory cases, escalation to immunosuppressants, such as mycophenolate mofetil or infliximab, may be necessary [41,160,174,175,176,177].

Early recognition and prompt multidisciplinary management, including dermatologic consultation, are essential to minimize morbidity and allow for potential rechallenge with immunotherapy in appropriate cases. Notably, the development of cutaneous irAEs has been positively correlated with improved oncologic outcomes, potentially serving as a surrogate marker of treatment efficacy in select patient populations [161,175,178,179,180,181].

**Figure 8:**
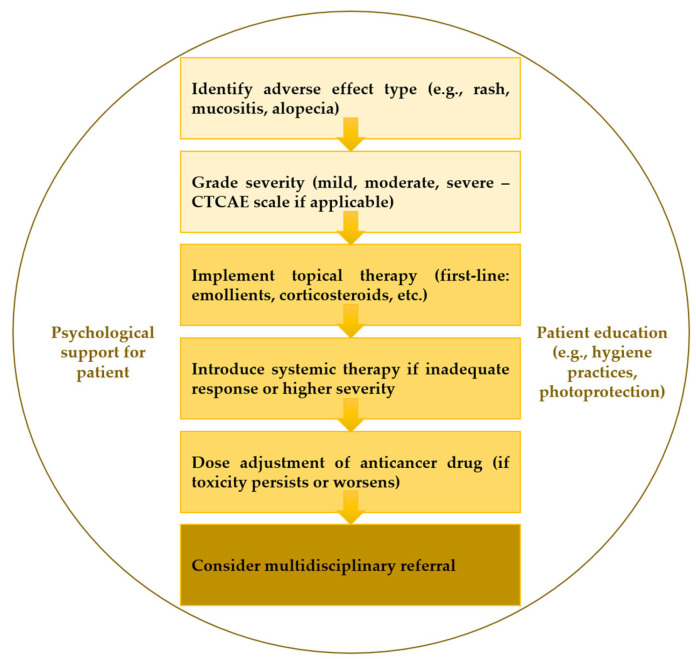
Stepwise management algorithm for dermatologic toxicities in breast cancer therapy (an original scheme based on the current literature).

The most prominent cutaneous adverse events in breast cancer therapy are shown in Table 1. This is followed by Table 2, which summarizes the treatment strategies tailored to specific adverse effects. A structured management algorithm is also provided in Figure 8 to guide clinicians from diagnosis to intervention.

## 7. Oral Mucosal Toxicities in Breast Cancer Patients

Oral mucosal toxicities represent a significant but frequently underrecognized component of systemic oncologic therapy in breast cancer. These adverse events may arise from cytotoxic chemotherapy, targeted agents, immunotherapies, and endocrine treatments, potentially compromising the patients’ quality of life, nutritional intake, and adherence to therapy [182]. Despite advances in cancer care, oral complications often receive less attention than dermatologic or gastrointestinal toxicities, even though their impact on functional well-being and psychosocial health—especially among female patients—is profound [182,183,184].

Timely identification, routine oral evaluations, and evidence-based supportive care are essential for mitigating mucosal toxicities and maintaining treatment continuity. Table 3 provides an overview of the most prominent oral adverse events, their clinical features, causative agents, and management strategies.

Oral mucositis is the most prevalent and clinically significant mucosal complication, particularly associated with agents such as 5-fluorouracil (5-FU), capecitabine, and methotrexate [185]. It typically presents as erythema, ulceration, and severe pain, often leading to impaired oral intake and compromised oral hygiene. The incidence in breast cancer patients ranges from 40 to 55%, depending on the regimen used [186,187]. Clinically, mucositis follows a stereotyped progression from erythema to ulceration and may be complicated by secondary infection. Notably, the severity of pain may exceed the visible extent of mucosal damage, necessitating prompt analgesic and nutritional support [188,189].

Stomatitis, particularly aphthous-like ulcerations, frequently occurs with mechanistic target of rapamycin (mTOR) inhibitors such as everolimus. Lesions often develop within the first treatment cycle and may become dose-limiting. Prophylactic use of dexamethasone mouthwash has demonstrated significant efficacy in reducing the incidence and severity of everolimus-induced stomatitis and is now considered a standard supportive intervention [190,191,192,193].

Oral candidiasis is frequent in immunocompromised patients or those receiving immunosuppressive regimens [194]. Xerostomia (dry mouth) and dysgeusia (altered taste perception) occur across multiple drug classes and can substantially impair nutrition, speech, and oral hygiene adherence [195,196,197]. These conditions not only diminish physical comfort but also affect social functioning and emotional well-being.

The development of oral mucosal reactions is multifactorial, involving direct cytotoxic effects on rapidly dividing epithelial cells, alterations in local immunity, inflammatory cytokine release, and disruptions to the oral microbiome [198,199]. Targeted therapies such as EGFR inhibitors impair epithelial homeostasis and regeneration, while mTOR inhibitors interfere with angiogenesis and mucosal repair [193,200]. Hormonal therapies may reduce salivary gland function, contributing to mucosal dryness and secondary infections [201]. Host-related factors, including age, comorbidities, oral hygiene status, baseline mucosal health, and genetic predisposition, also modulate the susceptibility and severity of mucosal toxicity [202,203]. In addition, recent studies have suggested that the diversity and composition of the oral microbiota may influence both the severity and healing trajectory of mucosal lesions [204,205]. Disruption of microbial homeostasis may also predispose patients to opportunistic infections, particularly candidiasis and further oral complications such as xerostomia [194,206]. Importantly, shifts in oral microbial profiles during cancer therapy have also been associated with systemic inflammatory responses, suggesting a potential two-way interaction between local toxicity and systemic treatment tolerance [207].

A systematic and proactive approach is crucial in managing patients at risk of or presenting with mucosal toxicities. This includes baseline oral evaluation prior to the initiation of cancer therapy including dental hygiene status, prosthetic devices, and identification of pre-existing lesions; regular follow-up examinations during treatment, especially in the first two to four weeks of new regimens; and the grading of mucosal toxicity using validated scales such as the CTCAE; and microbiological evaluation when signs of secondary infection are present, especially with candidiasis or ulceration unresponsive to supportive measures [41,208]. Close interdisciplinary communication between oncology, dental medicine, and supportive care providers is essential for the rapid adjustment of interventions and minimization of treatment delays. Additionally, digital tools and photographic monitoring may aid in objective lesion tracking, especially in outpatient settings. Integrating oral health professionals into the oncology care team has been shown to improve both symptom control and patient satisfaction, highlighting the value of a truly multidisciplinary approach [209,210].

Preventive strategies may be essential and include rigorous oral hygiene measures with non-irritating, alcohol-free rinses; cryotherapy during 5-FU infusion, which has shown significant reduction in mucositis severity; and dexamethasone mouthwash prophylaxis during mTOR inhibitor therapy, which reduces the risk of severe stomatitis [106,190,191,193,211]. Patient education on oral care and early symptom reporting is also critical. Treatment of established mucosal toxicities includes topical analgesics (e.g., lidocaine), anti-inflammatory agents (e.g., topical corticosteroids), and antifungals (e.g., nystatin, fluconazole) depending on etiology as well as systemic analgesics and nutritional support in cases of severe pain or weight loss [193,194,202,208]. Maintaining the prescribed cancer treatment schedule is of paramount importance, as interruptions or dose reductions due to oral toxicities can compromise the therapeutic efficacy and overall survival outcomes. Effective symptom control and early intervention are therefore essential to allow for uninterrupted oncologic therapy. Where necessary, interventions are encouraged to be rapid, targeted, and reversible to ensure that cancer treatment may proceed with minimal modification.

In breast cancer management, oral toxicities may result not only from traditional cytotoxic agents, but also from targeted drugs such as everolimus, trastuzumab-deruxtecan, or long-term endocrine therapy [193,201,212,213]. The latter may cause chronic xerostomia and mucosal atrophy, necessitating ongoing oral care even beyond active treatment [201]. Notably, mucositis associated with trastuzumab-deruxtecan has been reported, although less frequently than with other agents [212,213]. In long-term survivors, chronic mucosal complications may persist and require dedicated follow-up protocols involving oral medicine specialists, underscoring the need for sustained oral health monitoring [201]. Research into probiotic therapy and oral microbiome modulation offers promise in mucositis prevention [214]. In addition to conventional preventive and therapeutic strategies, recent advances in nanotechnology offer promising avenues for mitigating mucocutaneous side effects associated with breast cancer therapy. Nanoformulated topical agents and targeted drug delivery systems are being developed to enhance therapeutic efficacy while reducing systemic toxicity [215]. These nanoparticle-based delivery platforms, including mucoadhesive nanogels, polymeric nanoparticles, liposomes, and solid lipid nanoparticles, enable the localized and sustained release of anti-inflammatory, analgesic, antifungal, or regenerative agents directly at the site of tissue injury. This targeted approach enhances drug penetration, improves local bioavailability, and minimizes off-target effects.

**Table 3:** Oral mucosal adverse effects of breast cancer therapies and their recommended management (an original table based on the current literature).

Therapy Type	Drug(s)	Oral Mucosal Adverse Effects	Severity (CTCAE)	Management Strategies	References
Chemotherapy	5-FU, capecitabine, methotrexate, trastuzumab-deruxtecan	Oral mucositis (pain, erythema, ulcers)	Grade 1–3	Cryotherapy during 5-FU, bland rinses, topical lidocaine, systemic analgesics	[185,186,187,188,189,193,194,202,208,212,213]
Targeted therapy	e.g., everolimus	Stomatitis (aphthous-like lesions)	Grade 1–2	Dexamethasone mouthwash (prophylactic), topical corticosteroids	[106,190,191,192,193,211]
Chemotherapy/immunotherapy	Any immunosuppressive therapy	Oral candidiasis	Grade 1–2 (usually)	Topical antifungals (nystatin), systemic fluconazole, oral hygiene	[194]
Hormonal therapy	Aromatase inhibitors	Xerostomia, mucosal atrophy	Grade 1	Saliva substitutes, oral moisturizers, frequent hydration	[201,204,205]
EGFR/mTOR inhibitors	Trastuzumab-deruxtecan, everolimus	Dysgeusia, xerostomia, delayed healing	Grade 1–2	Zinc supplementation (optional), salivary stimulants, mucosal protective agents	[195,196,197]

For example, curcumin-loaded polymeric nanoparticles have demonstrated efficacy in preclinical models of chemotherapy-induced mucositis by modulating oxidative stress and inflammatory cytokine expression [215]. In a preliminary randomized clinical trial, curcumin nanomicelle capsules or mouthwash significantly reduced the incidence and severity of radiotherapy-induced oral mucositis in patients with head and neck cancer compared with the placebo controls [216]. Additionally, a recent systematic review in animal models demonstrated that gold nanoparticles (AuNPs) effectively reduced ulceration, inflammatory biomarkers, and oxidative stress in chemotherapy-associated oral mucositis [217]. Mucoadhesive nanosystems incorporating corticosteroids or analgesics have also been shown to provide localized and prolonged symptom control, further supporting their therapeutic potential.

Although most of these nanoformulations are still in the experimental or early clinical stages, they represent a novel and potentially transformative adjunct to standard supportive care. As such, nanotechnology-based interventions may become increasingly relevant, particularly in patients who are refractory to conventional management or at elevated risk for severe mucosal toxicity.

**Figure 9:**
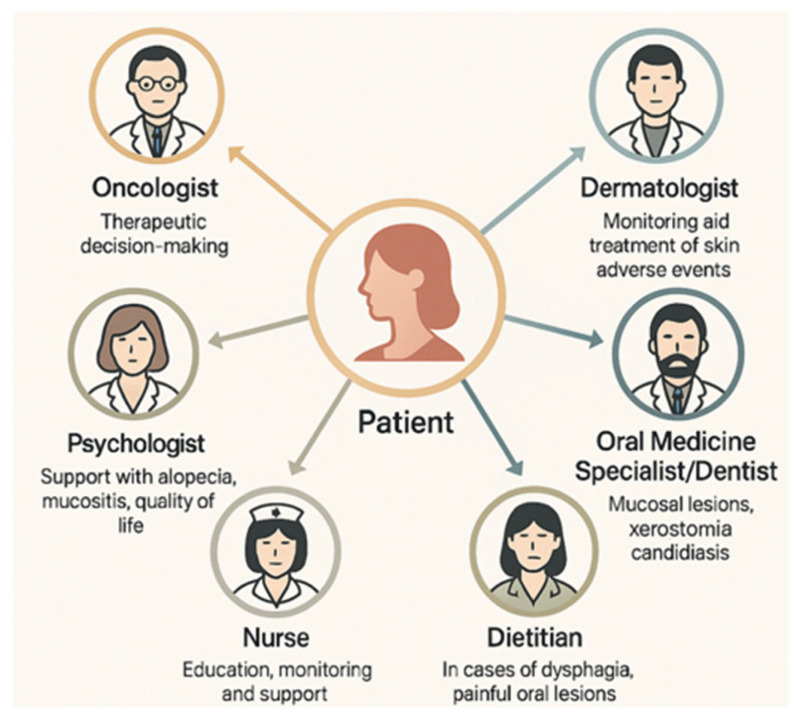
Multidisciplinary team (MDT) for managing cutaneous and oral mucosal toxicities in breast cancer (an original scheme based on current literature data).

Pharmacogenomic profiling may also help identify individuals predisposed to severe mucosal reactions, potentially guiding therapy selection and pre-emptive intervention [202,218]. Future clinical guidelines should incorporate patient-specific factors, including oral status, pharmacogenomic profile, and treatment goals, into risk-adapted mucosal care protocols. Such personalized approaches have the potential to reduce the toxicity burden and improve the overall cancer treatment outcomes. As breast cancer therapies become increasingly individualized, supportive care strategies and multidisciplinary teams (MDTs) are also advised to evolve (Figure 9), ensuring that treatment advances are matched by the equally progressive management of associated toxicities. Personalized approaches that incorporate molecular profiling, predictive biomarkers, and pharmacogenomics represent the next frontier in mitigating mucocutaneous adverse events and preserving treatment adherence in precision oncology.

## 8. Conclusions

This review provides a comprehensive and integrative overview of both dermatologic and oral mucosal toxicities associated with all major therapeutic modalities in breast cancer management. In contrast to the previous literature—often limited to specific drug classes or dermatologic reactions—this article introduced a unified clinical framework encompassing chemotherapy, endocrine therapy, targeted agents, immunotherapy, and radiotherapy. Notably, we highlighted emerging strategies, including nanoparticle-based drug delivery systems, predictive pharmacogenomics, and interdisciplinary care models, reflecting the dynamic evolution of precision oncology.

Cutaneous adverse effects (CAEs) are among the most common complications of contemporary cancer therapy. These toxicities span a broad clinical spectrum—from mild xerosis and rash to more debilitating manifestations such as alopecia, hand-foot syndrome, and radiodermatitis. Although often distressing due to their visibility and impact on body image, most CAEs are preventable or reversible with early recognition, patient education, and evidence-based interventions. Routine skincare, sun protection, and pharmacologic support form the cornerstone of prevention and symptom control.

As treatment regimens become increasingly complex and multimodal, the diversity and incidence of mucocutaneous toxicities are expected to rise. This necessitates a multidisciplinary and anticipatory approach involving oncologists, dermatologists, oral medicine specialists, and supportive care teams to ensure the prompt identification, appropriate intervention, and continuity of cancer therapy. Importantly, the psychological burden of visible mucocutaneous side effects must not be overlooked, as it can negatively influence treatment adherence, quality of life, and patient satisfaction.

In conclusion, mucocutaneous toxicities, while often unavoidable, need not represent an insurmountable barrier to successful breast cancer treatment. With proactive management, individualized care pathways, and holistic patient support, these adverse effects can be effectively mitigated—allowing for the preservation of oncologic efficacy and the enhancement of patient-centered outcomes.

## Figures and Tables

**Table 1:** The most prominent cutaneous adverse events in breast cancer therapy with the severity, frequency, pathophysiology, and management strategies (an original table based on the current literature).

Therapy Type	Drug(s)	Cutaneous Adverse Effects	Severity (CTCAE)	Frequency	Mechanism/Pathophysiology	Management Strategies	References
Chemotherapy	Paclitaxel, Docetaxel	Alopecia, nail changes, xerosis	Grade 1–2	Very common	Targets rapidly dividing matrix keratinocytes; affects skin adnexa	Scalp cooling, minoxidil 5%, emollients, nail protection	[16,18,19,20,21,22,23,24,25,26,27,28,29,30,31,32,33,34,35,36,52,53,54,55,56,57,58,59,60,61,62,63,64,65,66,67]
Chemotherapy	Capecitabine, Doxorubicin (lip.)	Hand-foot syndrome	Grade 1–3	Common	Cytotoxicity to basal epidermal keratinocytes in high-pressure areas	Urea creams, corticosteroids, analgesics, dose reduction	[37,38,39,40,41,42,43,44,45]
Targeted therapy	Lapatinib	Acneiform rash, pruritus, xerosis	Grade 1–2	Common	EGFR inhibition disrupts follicular keratinocyte differentiation and immune homeostasis	Tetracyclines, topical corticosteroids, moisturizers	[63,97,98,99,100,101,102]
Targeted therapy	Alpelisib	Maculopapular rash	Grade 1–3	Common	PI3K pathway inhibition → cytokine-mediated inflammation	Antihistamines, topical/systemic corticosteroids, therapy interruption	[103,104,105,106,107,108]
Hormonal therapy	Anastrozole, Tamoxifen	Alopecia, urticarial vasculitis, xerosis	Grade 1–2	Common	Estrogen depletion alters epidermal turnover and hair cycling; immune-mediated reactions	Minoxidil, corticosteroids, hydration therapy	[63,117,118,119,120,121,122]
Radiotherapy	N/A	Radiodermatitis	Grade 1–3	Very common	Radiation-induced damage to basal keratinocytes and skin vasculature	Hyaluronic acid, silver sulfadiazine, topical corticosteroids	[133,134,135,136,137,138,139,140,141,142,143,144,145,146,147,148,149,150]
Immunotherapy	Atezolizumab	Lichenoid rash, Vitiligo-like depigmentation	Grade 1–3	Less common	T-cell-mediated autoimmune reaction triggered by immune checkpoint blockade	Topical/systemic corticosteroids, immunotherapy suspension if necessary	[151,152,153,154,155,156,157,158,159,160,161,162,163,164,165,166,167,168,169,170,171,172,173,174,175,176,177,178,179,180,181]

**Table 2:** An overview of the cutaneous adverse effects of breast cancer therapies and recommended treatment strategies (an original table based on the current literature).

Adverse Effect	Causative Agents/Drug Classes	Recommended Topical Treatment	Recommended Systemic Treatment	References
Alopecia	Taxanes (paclitaxel, docetaxel), anthracyclines, aromatase inhibitors, tamoxifen	Topical minoxidil, calcitriol, bimatoprost (eyelashes); scalp cooling during chemotherapy	Low-dose oral minoxidil (LDOM), spironolactone	[18,19,20,21,22,23,24,25,26,27,28,29,30,31,32,33,34,35,36,120,121]
Nail changes	Taxanes, EGFR inhibitors, anthracyclines	Emollients, nail strengtheners, topical antibiotics, and corticosteroids; cooling gloves	Oral antibiotics (e.g., doxycycline), analgesics	[16,52,53,54,55,56,57,58,59,60,61,62,63,64,65,66,67]
Erythema exudativum multiforme	Platinum-based drugs, antimetabolites	Topical corticosteroids, emollients	Systemic corticosteroids	[46,47,48,49,50,51]
Xerosis	EGFR inhibitors, hormonal therapy	Soap-free cleansers (pH 5–6), moisturizers with ceramides, niacinamide, 3–10% urea cream	–	[16,61,62,63,64,97,98,122]
Hand-foot syndrome (HFS)	Capecitabine, liposomal doxorubicin, 5-FU	Urea 10% cream, high-potency corticosteroids, analgesic patches	COX-2 inhibitors, pyridoxine (B6), analgesics	[37,38,39,40,41,42,43,44,45]
Acneiform eruptions	EGFR inhibitors (lapatinib), trastuzumab	Topical antibiotics, corticosteroids, retinoids, benzoyl peroxide	Oral tetracyclines (e.g., doxycycline)	[63,97,98,99,100,101,102]
Photosensitivity	Methotrexate, 5-FU, dacarbazine	Topical corticosteroids, broad-spectrum sunscreens	Oral corticosteroids, analgesics	[68,69]
Recall reactions	Methotrexate, gemcitabine, doxorubicin	Topical corticosteroids, wound care	–	[52,54,74]
Skin necrosis	Doxorubicin (extravasation), vinca alkaloids	Wound care	–	[61,75,76,77]
Neutrophilic eccrine hidradenitis	Cytotoxic chemotherapy (e.g., cytarabine, bleomycin)	–	Systemic corticosteroids, dapsone, analgesics	[78,79]
Eccrine squamous metaplasia	Cytotoxic chemotherapy	–	Systemic corticosteroids, dapsone, analgesics	[80,81,82,83]
Hyperpigmentation	Bleomycin, 5-FU, daunorubicin, EGFR inhibitors	Bleaching agents (e.g., hydroquinone), emollients	Oral antihistamines (for pruritus)	[8,70,71,72,73]
Sclerotic dermal reactions	Radiotherapy, taxanes, gemcitabine	High-potency corticosteroids, tacrolimus, imiquimod, emollients	Systemic corticosteroids	[84,85,86,87,88]
Raynaud’s phenomenon	Tamoxifen, cisplatin	–	Calcium channel blockers, ACE inhibitors	[89,90,91]

## Data Availability

Not applicable.

## Abbreviations

The following abbreviations are used in this manuscript:

5-FU	5-Fluorouracil
ACE	Angiotensin-converting enzyme
(C)AEs	(Cutaneous) adverse events
AIs	Aromatase inhibitors
AuNPs	Gold nanoparticles
CDK4/6	Cyclin-dependent kinase 4 and 6
CIA	Chemotherapy-induced alopecia
COX-2	Cyclooxygenase-2
CTCAE	Common Terminology Criteria for Adverse Events
DHEA	Dehydroepiandrosterone
EEM	Erythema exudativum multiforme
EGFR	Epidermal growth factor receptor
GSM	Genitourinary syndrome of menopause
HER2	Human epidermal growth factor receptor 2
HFS	Hand-foot syndrome
ICIs	Immune checkpoint inhibitors
IrAEs	Immune-related adverse events
LDOM	Low-dose oral minoxidil
MDT	Multidisciplinary team
mTOR	Mechanistic target of rapamycin
NEH	Neutrophilic eccrine hidradenitis
NSAIDs	Nonsteroidal anti-inflammatory drugs
PI3Kα	Phosphoinositide 3-kinase alpha
PI3K–AKT–mTOR	Phosphoinositide 3-kinase–AKT–mechanistic target of rapamycin pathway
PIK3Cα	Phosphatidylinositol-4,5-bisphosphate 3-kinase catalytic subunit alpha
RTOG	Radiation Therapy Oncology Group
SCLE	Subacute cutaneous lupus erythematosus
SERMs	Selective estrogen receptor modulators
SJS	Stevens–Johnson syndrome
SPF	Sun protection factor
TNBC	Triple-negative breast cancer
UV	Ultraviolet
